# Trends in respiratory virus circulation following COVID-19-targeted nonpharmaceutical interventions in Germany, January - September 2020: Analysis of national surveillance data

**DOI:** 10.1016/j.lanepe.2021.100112

**Published:** 2021-06-07

**Authors:** Djin-Ye Oh, Silke Buda, Barbara Biere, Janine Reiche, Frank Schlosser, Susanne Duwe, Marianne Wedde, Max von Kleist, Martin Mielke, Thorsten Wolff, Ralf Dürrwald

**Affiliations:** a Unit 17: Influenza and Other Respiratory Viruses | German National Influenza Center, Department of Infectious Diseases, Robert Koch-Institute, D-13353 Berlin, Germany; b The Rockefeller University, New York, NY, United States; c Department of Infectious Diseases Epidemiology, Robert-Koch Institute, Germany; d Computational Epidemiology (P4), Robert Koch-Institute, Germany; e Institute for Theoretical Biology, Humboldt University of Berlin, D-10115 Berlin, Germany; f Systems Medicine of Infectious Disease (P5), Robert Koch-Institute, Germany; g Department of Infectious Diseases, Robert Koch-Institute, Germany

**Keywords:** Nonpharmaceutical interventions, SARS-CoV-2, Respiratory virus, Rhinovirus, Surveillance

## Abstract

**Background:**

During the initial COVID-19 response, Germany's Federal Government implemented several nonpharmaceutical interventions (NPIs) that were instrumental in suppressing early exponential spread of SARS-CoV-2. NPI effect on the transmission of other respiratory viruses has not been examined at the national level thus far.

**Methods:**

Upper respiratory tract specimens from 3580 patients with acute respiratory infection (ARI), collected within the nationwide German ARI Sentinel, underwent RT-PCR diagnostics for multiple respiratory viruses. The observation period (weeks 1-38 of 2020) included the time before, during and after a far-reaching contact ban. Detection rates for different viruses were compared to 2017-2019 sentinel data (15350 samples; week 1-38, 11823 samples).

**Findings:**

The March 2020 contact ban, which was followed by a mask mandate, was associated with an unprecedented and sustained decline of multiple respiratory viruses. Among these, rhinovirus was the single agent that resurged to levels equalling those of previous years. Rhinovirus rebound was first observed in children, after schools and daycares had reopened. By contrast, other nonenveloped viruses (i.e. gastroenteritis viruses reported at the national level) suppressed after the shutdown did not rebound.

**Interpretation:**

Contact restrictions with a subsequent mask mandate in spring may substantially reduce respiratory virus circulation. This reduction appears sustained for most viruses, indicating that the activity of influenza and other respiratory viruses during the subsequent winter season might be low,whereas rhinovirus resurgence, potentially driven by transmission in educational institutions in a setting of waning population immunity, might signal predominance of rhinovirus-related ARIs.

**Funding:**

Robert Koch-Institute and German Ministry of Health.

**RESEARCH IN CONTEXT**

**Evidence before this study**

Nonpharmaceutical interventions (NPIs) introduced in early 2020 during the first wave of COVID-19 have appeared to affect the spread of infections with respiratory viruses other than SARS-CoV-2. This became first apparent when influenza activity decreased substantially during Southern hemisphere winter. Virological surveillance data characterizing the effect of NPIs on respiratory viruses other than influenza at the national level is still scarce.

**Added value of this study**

The laboratory-based virological surveillance program at the German National Influenza Centre monitors the circulation of multiple respiratory viruses, including human rhinovirus (HRV), an agent that remains unconsidered in many sentinel studies. Molecular diagnostic data on over 15000 specimens from 2017-2020 indicates the NPIs implemented during the early German COVID-19 response decreased the activity of all established respiratory viruses in an unprecedented and prolonged fashion. The single virus that rebounded to the levels of previous years was human rhinovirus.

**Implications of all the available evidence**

This data indicates that nonpharmaceutical interventions, including a temporary strict contact ban and the use of face masks, may be effective at lowering respiratory viral disease burden. Although it is difficult to disentangle the contribution of specific interventions and certain confounders can not be entirely ruled out, the public health measures implemented to curb the spread of COVID-19 in 2020 may have profoundly impacted even the subsequent winter season, potentially conditioning low influenza but consistent rhinovirus activity.

Alt-text: Unlabelled box

## 1 Introduction

Acute respiratory viral infections are an important cause of morbidity and mortality worldwide, especially in vulnerable individuals. Virological surveillance of respiratory infections, ideally done in sentinel studies that are based on a statistically representative selection of geographically distinct clinics, is key to monitoring the prevalence, seasonal patterns and genetic diversity of the causative agents. Pharmaceutic treatment options for most respiratory viruses remain limited, which renders particular importance to public health measures that prevent their spread, namely nonpharmaceutical interventions (NPIs) [1,2][4], [5]. Prior to the COVID-19 pandemic, the impact of NPIs, implemented on a nationwide scale, on the circulation of respiratory viruses has been vastly unknown. During the first wave of the pandemic, it emerged that NPIs impacted not only SARS-CoV-2 but also influenza virus [3,4] with a pronounced decline of influenza activity during Southern hemisphere winter [5], [6], [7]; and that pediatric hospitalizations due to non-COVID-19 respiratory illnesses decreased substantially [8,9]. However, national-level lab-based surveillance data characterizing the effect of NPIs on multiple respiratory viruses is currently scarce to the best of our knowledge.

The first German COVID-19 cases, detected in late January, were successfully contained [10,11] and the general consensus is that the pandemic arrived later in Germany than in many other European countries. However, several clusters, related to returning travelers and local carnival celebrations were formed in February and resulted in epidemic spread of SARS-CoV-2. In an effort to counter the exponential rise of cases, the German Federal Government deployed a series of concerted NPIs, resulting in a notable reduction of the SARS-CoV-2 spreading rate [1]. Here we describe how the restrictions suppressed not only the spread of SARS-CoV-2 but also that of other viral pathogens, based on 2017-2020 virological data from Germany's national sentinel system for monitoring ARIs, particularly influenza.

## 2 Methods

### 2.1 Virological sentinel

**Fig. 1:**
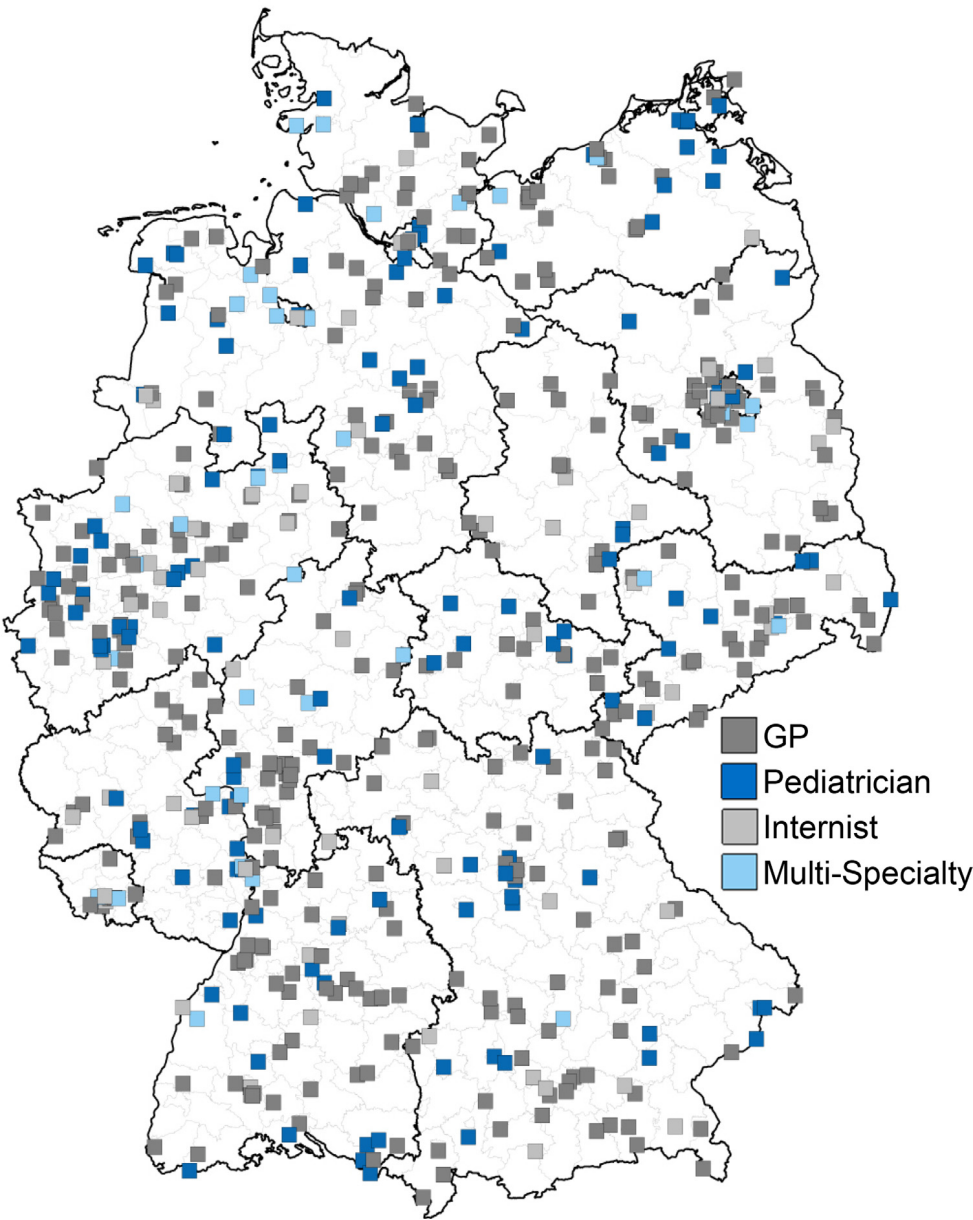
**Spatial Distribution of Sentinel Clinics over Germany.** Shown are the geographical locations of clinics participating in the national sentinel system for monitoring acute respiratory infections (ARI), particularly influenza. High population density is reflected in high practice density. Different colors represent different specialties. GP, General Practioner.

Laboratory-based virological surveillance is a key instrument in Germany's national sentinel system for monitoring acute respiratory infections (ARI), particularly influenza^1^. Figure 1 shows the geographical distribution of sentinel clinics over the entire German territory. More than 1 % of primary care physicians participate in the sentinel and thus over 1% of the population is represented [12,13]. Approximately 20% of sentinel physicians in geographically representative practices nationwide are asked to systematically sample ambulatory patients presenting with ARI / influenza-like illness, prioritizing those with fever or other systemic signs of illness [14]. The ARI case definition (acute respiratory disease with at least one of the four following symptoms: fever, cough, rhinorrhoea or sore throat) has been maintained throughout, even after COVID 19 became a pandemic. Sentinel physicians collect upper respiratory specimens (mostly, nasal or pharyngeal swabs) from ambulatory patients. Specimens undergo molecular diagnostics at the German National Influenza Center, where presence of influenza virus A/B (IV), human respiratory syncytial virus (RSV), metapneumovirus (HMPV), rhinovirus (HRV), parainfluenzavirus (PIV, since 2020) and SARS-CoV-2 (since 2020) are routinely assessed, as outlined in the section *Laboratory analyses* below. Thus, lab-based surveillance for influenza, a notifiable illness, is complemented with lab-based surveillance for a range of non-notifiable viral respiratory diseases [12,[15], [16], [17]]. This virological sentinel enables monitoring the spread of a broad spectrum of respiratory RNA viruses at the national level; respiratory RNA viruses have public health relevance because they belong to viral families that are considered to have potential to trigger pandemics [18]. *Sample Numbers:* The sentinel is designed so that during the summer season, when between 30 and 100 samples per week are analyzed, there is a 95% probability that viruses are detected with prevalences among tested individuals exceeding 3% (100 samples) -12% (30 samples). Conversely, if we do not detect a virus we can state that its true prevalence lies with 95% confidence within the interval 0 – 11.35% (30 samples/week) and 0 – 3.70% (100 samples/week). As ARI activity increases during each winter season, higher sample numbers are collected by the sentinel physicians and more samples undergo lab analysis, usually ranging from 100 at the very minimum to ca. 400 samples per week. Higher numbers of samples examined result in higher sensitivity of the sentinel, allowing us to detect emerging outbreaks at a lower prevalence and therefore earlier (see also Supplementary Figure S1).

### 2.2 Ethics statement

Written and informed consent was obtained from all sentinel patients. All investigations were conducted according to the principles expressed in the Helsinki Declaration. Written approval for the German national surveillance of influenza and other respiratory viruses was obtained from the Charité-Universitätsmedizin Berlin Ethical Board (reference EA2/126/11) and sentinel surveillance is covered by German legislation (§13, §14, Protection against Infection Act). All analyses were based on pseudonymised data.

### 2.3 Laboratory analyses

#### 2.3.1 Sample preparation, viral RNA extraction and c-DNA synthesis

Sample preparation, viral RNA extraction and c-DNA synthesis were performed as previously described [12,[15], [16], [17],^19^]. Briefly, nasal or oropharyngeal swabs(Copan Diagnostics, Murrieta, United States) were sent to the National Influenza Centre, where 3 mL of cell culture medium [minimum essential medium (MEM) with N-2-hydroxyethylpiperazine-N-2-ethane sulfonic acid (HEPES) buffer with 5,000U/mL PenStrep)] were added to wash out the attached viruses. RNA was extracted from 200 µL sample material, employing the MagNA Pure 96 DNA and Viral NA Small Volume Kit (Roche, Mannheim, Germany) or the MagNA Pure 24 Total NA Isolation Kit and eluting in 50 µL buffer. For c-DNA synthesis in a total volume of 40 µL, 25µL RNA, random hexamer primers and 200U Moloney murine leukaemia virus (M-MLV) Reverse Transcriptase (Thermo Fisher Scientific, Waltham, US) underwent reverse transcription under the following thermocycling conditions: 42°C (5 min), 37°C (30 min) and 95°C (5 min). c-DNA was diluted 1:1 with H_2_O for downstream PCR assays.

#### 2.3.2 Modular real-time PCR assays for detection of respiratory viral pathogens

For molecular diagnostic detection of IV-A, IV-B, RSV, HRV, HMPV, PIV, SARS-CoV-2 and FCV (feline calicivirus, which serves as an internal process control), a real-time PCR system that is modular in design, allowing to run reactions in singleplex or multiplex formats, is established at the National Influenza Centre. This system includes previously described real-time PCR assays [12,[15], [16], [17],^19^,^20^][6], which were (i) modified to take new insights into genetic variation into account and (ii) adapted to and extensively validated for the modular design, where single- and multiplex reactions can be run under similar reaction conditions. PCRs were performed on LC480II real-time PCR thermal cyclers (Roche, Basel, Switzerland) in 96- or 384-well plates. Each reaction contained, in a total volume of 20µL, 1x PCR buffer, 4mmol/L MgCl2, 1mmol/L deoxynucleoside triphosphate (dNTP; Thermo Fisher Scientific, Waltham, US) with deoxyuridine triphosphate (dUTP; GE Healthcare, Chicago, US), 600ng bovine serum albumin (BSA; Thermo Fisher Scientific, Waltham, US), 0.3U (singleplex) or 1U (multiplex) Platinum Taq Polymerase (Thermo Fisher Scientific, Waltham, US), oligonucleotides as listed in Supplementary Table S1 (Metabion, Planegg, Germany and Applied Biosystems, Foster City, USA), and 5µL of prediluted c-DNA. Thermocycling parameters were as follows: 5 min at 95 °C for Taq DNA polymerase activation and initial denaturation prior to a total 45 cycles consisting of denaturation at 95 °C for 15 s and annealing at 60 °C for 30 s. Data was analysed using the LightCycler software version 1.5.1.

### 2.4 Statistical analyses

To assess whether the frequencies of respiratory viruses observed in each week of 2020 were significantly lower than in the preceding years, we assumed that the detection of any respiratory virus follows a binomial distribution. One-sided P-values (P_20vsX) were determined accordingly (binomial test), to test whether there were significantly less respiratory viruses in 2020 compared to X = 2017, -18, -19 respectively. The combined P-value for assessing whether respiratory virus detections in 2020 were significantly lower than in all of the previous years 2017-19 was then computed as:

P-Value = 1-[(1-Pvalue_20vs.17)*(1-Pvalue_20vs.18)*(1-Pvalue_20vs.19)].

In addition, Pvalue_20vs.17, Pvalue_20vs.18 and Pvalue_20vs.19 were determined using the Fisher Exact test and combined P-values were calculated using an analogous approach.

### 2.5 Changes in mobility

Based on recent work indicating that mobility may be used as an indicator for the strength of lockdown measures in multiple countries (i.e. the rate with which social contacts are effectively reduced [21] and a proxy indicator for the adoption rate of other non-compulsory and non-medical interventions [22], mobility may be considered as an indicator for changes in the population that affect the infection dynamics [23]. Therefore, 2020 mobility data is displayed in order to provide additional context for the 2020 respiratory virus surveillance data. To this end, changes in mobility were calculated based on mobility flows collected from mobile phone data: The total number of trips in a given 2020 week was compared to the total number of trips during the corresponding week in 2019. Mobility change calculations have been described in considerable detail elsewhere [21,24].

### 2.6 Use of disease notification data

To complement findings from the virological surveillance with information on the circulation of non-respiratory viruses, which are not covered by our lab-based surveillance, we used data from the system for surveillance of notifiable infectious diseases. Briefly, the Protection Against Infection Act determines which infectious diseases (§6, notifications by medical doctors) and which detected pathogens (§7, notifications by laboratories) are notifiable in Germany; for each notifiable disease case definitions exist that involve epidemiological, clinical and laboratory criteria [25,26]. The SurvStat@RKI 2.0 online tool [27], was used on November 9, 2020 to provide aggregated data regarding seasonal influenza, norovirus gastroenteritis and rotavirus gastroenteritis reported to local or federal public health authorities between week 1, 2017 and week 38, 2020.

### 2.7 Role of the funding source

These investigations were funded by Robert Koch-Institute and German Ministry of Health. The authors had sole responsibility for the design and execution of the study, the collection, analysis and interpretation of data and the preparation of the manuscript.

## 3 Results

### 3.1 Timeline of NPIs

Governmental NPIs were introduced in a stepwise fashion, beginning with cancellation of mass gatherings, which was followed by closure of schools, cultural venues and many businesses (week 11-12; table 1). These physical distancing measures were increased in week 13, when a strict, extensive contact ban was announced, which included the prohibition of gatherings of people from different households and the closure of all nonessential businesses. These interventions were accompanied by marked decreases in population mobility [21].

**Table 1:** Governmental NPIs and surrounding events during the early German COVID-19 response

Date	Calendar week	Intervention
March 9-12, 2020	11	Mild Physical Distancing: Cancellation of mass gatherings, e.g. trade fairs and soccer games Calls to avoid social gatherings Population mobility starts to decline.
March 16-18, 2020	12	Strong Physical Distancing: Closure of schools, childcare facilities, many businesses and cultural venues incl. bars / clubs. Nonessential international travel ban
March 23, 2020	13	Strict contact ban: Prohibition of small gatherings of people not from the same household Closing of all nonessential businesses Population mobility reaches nadir.
April 20, 2020	17	Federal Government begins process of easing restrictions gradually, starting with the reopening of small stores
April 27, 2020	18	Mask mandate in public spaces
May 6, 2020	19	Control of easing restrictions is transitioned from Federal Government to state governments
May 18, 2020	21	Many states begin stepwise reopening of schools and daycare centers for limited operations
June 15, 2020	25	Population mobility back up (>95% of 2019 levels).
Jul. 27- Aug. 8, 2020	31-32	Schools in almost all states closed for summer vacation

Careful lifting of restrictions began four weeks later, initially following a rather uniform approach determined by the federal government; and then in more heterogeneous ways, which were determined by each *Bundesland* (state) separately and thus varied state-to-state. School and day-care center operations were gradually resumed, depending on geography, beginning week 21, returning to almost normal conditions by weeks 23-27, just prior to summer vacation (the start date of which also varies by geography). In addition, most venues and businesses were allowed to reopen. Eased restrictions were reflected in increasing mobility, which was almost back at 2019 levels from week 25 on [21]. A mandatory mask requirement in public spaces, effective week 18, was retained throughout, as were physical distancing rules and hygiene measures introduced in the very early phase of the German epidemic.

### 3.2 Respiratory virus surveillance results in the context of NPIs

**Fig. 2:**
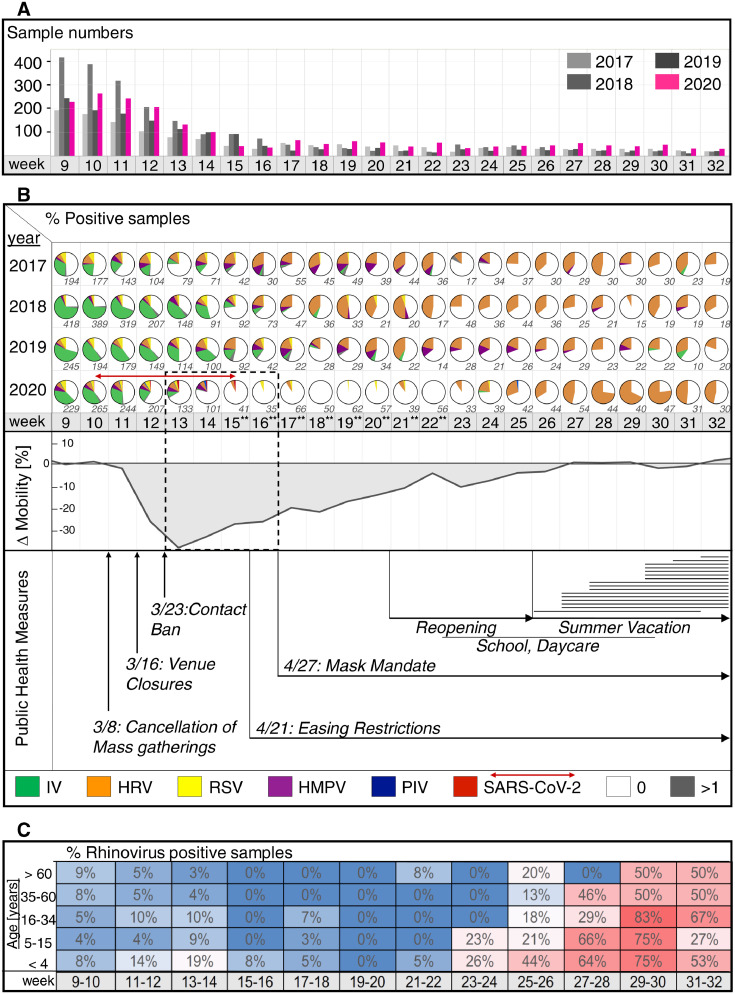
**Respiratory virus surveillance results in the context of public health measures.** Please note the complementary figures S2, S3 and S4 in the Supplementary section. **A. Specimen numbers per sampling week/ year.** Each column represents the numbers of samples obtained from patients presenting with acute respiratory illness in the calendar week indicated. Shades of grey / pink indicate the sampling year. **B. 2020 sentinel prevalence of respiratory viruses in the temporal context of NPIs and mobility, compared to 2017-2019 sentinel prevalence.** Areas of colored segments in each piechart represent the detection prevalence of a respiratory virus in samples obtained during the indicated week / year. Included is a 2020 mobility chart, displaying the relative change [%] in population mobility as compared to the corresponding 2019 week; an overview of NPIs; and summer vacation periods, which vary by region: each horizontal staggered line represents the vacation block of one *Bundesland* (state). Asterisks denote statistical significance level of 2020 respiratory virus prevalence being lower than in 2017-2019: **, p<0.005 based on both binomial and Fisher's exact tests. IV: Influenza A/B, HRV: Human Rhinovirus, RSV: Respiratory Syncytial Virus, HMPV: Human Metapneumovirus, PIV: Parainfluenzavirus, SARS-CoV-2: SARS Coronavirus 2, 0: negative for the tested viruses, >1: more than 1 virus detected. Both PIV and SARS-CoV-2 were only tested for in 2020. **C. Rhinovirus rebound by age group.** Heatmap diagram showing the percentage of rhinovirus-positive specimens by age group (Y-axis) and time (X-axis); time scale corresponds to two-week-blocks in 2020.

Specimens from 3580 patients with ARI symptoms, obtained by sentinel physicians between January and September, 2020, were sent to the National Influenza Center for lab-based respiratory virus surveillance, using multiplex PCR analysis. Specimen numbers and diagnostic results were compared to data of corresponding weeks in the three previous years (2017-2019; Table 2 and Supplementary Table S1). Sample counts displayed unusual re-increase starting week 10, and generally exceeded 2018-2019 counts starting week 17 (Fig. 2a and Suppl. Fig. S2b)^2^. SARS CoV-2 detection prevalence reflected national COVID-19 incidence well, with highest prevalence (3.1%) noted in samples collected in week 13, while 7-day-incidence of reported cases peaked at 36064 in week 14^3^. NPIs culminated in the contact ban effective week 13, which is when sentinel detection prevalence began to decline. From week 16 on, sentinel samples remained SARS-CoV-2 negative, indicating sustained lowering of SARS-CoV-2 epidemic activity (Fig. 2b and Suppl. Fig. S2b)[2].

**Table 2:** Virological surveillance 2017-2020: Specimens and viral pathogen distribution (weeks 1-38)

Year	N	Virus detected							
		IV A/B	HRV	HMPV	RSV	PIV	SARS-CoV-2	≥2	none
**2017**	3876	1254	440	98	287	n/d	n/d	57	1740
**2018**	4797	2087	379	208	180	n/d	n/d	92	1851
**2019**	3150	1042	333	80	285	n/d	n/d	56	1354
**2020**	3580	836	495	189	152	43	12	54	1799

IV A/B, human influenza virus A/B; RSV, respiratory syncytial virus; HMPV, human metapneumovirus; HRV, human rhinovirus; PIV, parainfluenzavirus; SARS-CoV-2, SARS coronavirus 2; ≥2, more than one virus detected; n/d, not done .

NPIs appeared to be temporally associated with drastic declines in the sentinel prevalence of other respiratory viruses: the percent positive tests in the sentinel in 2020 in weeks 12 and 14-22 were substantially lower than in the corresponding weeks of all previous years. For weeks 14-22, this difference was highly statistically significant (Fig. 2b, Suppl. Figure S2b [2] and Supplementary Table S3). Specifically, a sharp decrease of the percent positive tests for influenza was observed subsequent to NPI implementation; the last positive specimen of the season was obtained in week 14, a week after the contact ban became effective. By contrast, during previous seasons, influenza virus had been present in samples through weeks 22 (2017), 18 (2018) and 21 (2019). The last HMPV-positive specimen was also from week 14; previously HMPV had been present through weeks 38 (2017, Suppl. Figure S2b [2]), 31 (2018), and 33 (2019). RSV sentinelprevalence fell below 2017-19 levels in week 14, two weeks after day-care closures, with only sporadic detections afterwards. Thus, NPI deployment was followed by earlier and/or more abrupt ends to the 2020 influenza, HMPV and RSV seasons (Fig. 2b and Suppl. Fig. S2b[2]).

Whereas restrictions were being lifted from week 17 on, respiratory virus activity in the sentinel practically ceased for eight weeks with few sporadic RSV, HRV and PIV detections only. No respiratory virus at all was detected in week 18 and week 22 samples. Rhinovirus detection rates in previous years had surged immediately after influenza season ended, but in 2020 they remained low through week 22.

### 3.3 Rhinovirus is the only virus to resurge

Beginning in week 23, there was a notable uptick in the detection prevalence of rhinovirus (Fig. 2b). Rhinovirus was the single sentinel virus to not display lasting suppression and reach pre-COVID-19 detection levels. From week 27 on, detection rates exceeded those of the previous years, with well over 60% of all samples testing positive in weeks 28-30. Rhinovirus rebound was first observed in children, about two weeks earlier than in older individuals (Fig. 2c; absolute numbers corresponding to these percentages are provided in Suppl. Fig. S4). A similar, albeit less pronounced, pattern of rhinovirus increasing in children before it increased in adults was also observed in the previous years (Suppl. Fig. S3 and Suppl. Fig. S4). In 2020, rhinovirus detection rates rose first while schools and daycare centers were being reopened and dipped mildly from week 31 on, when almost all states were on school vacation (Fig. 2b[2]).

### 3.4 Case numbers of other viral infections in the context of NPIs

**Fig. 3:**
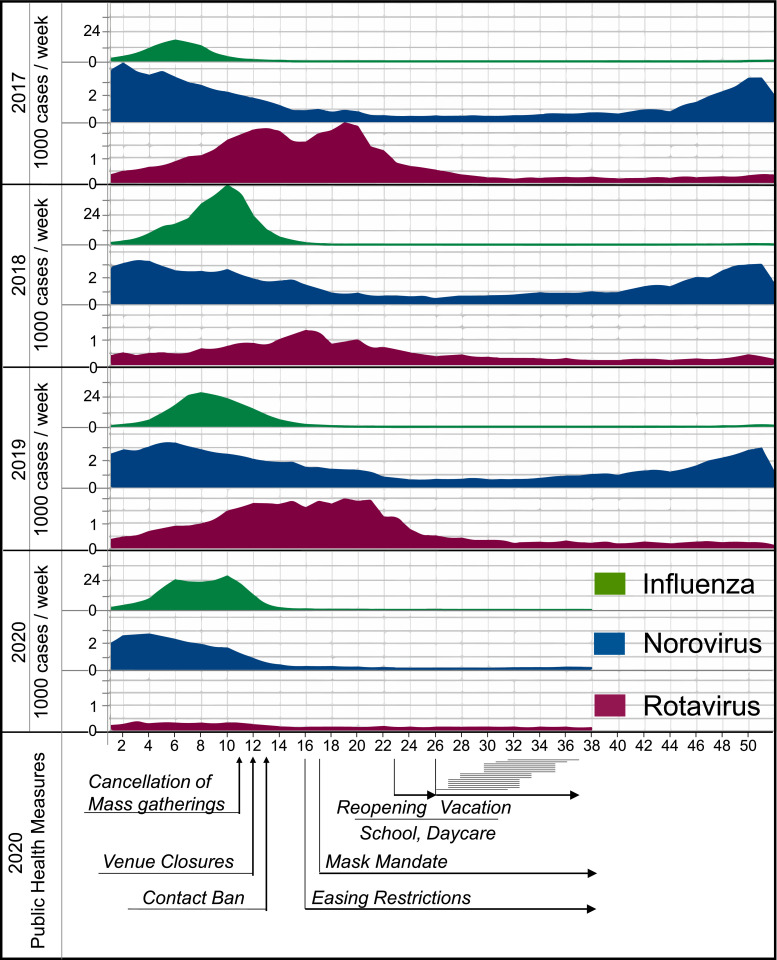
**Sustained decrease of case numbers for viral infections in 2020 and its temporal association with public health measures.** Shown is the number of cases reported to local or federal health authorities in 2017-2020 for each of three notifiable viral diseases: seasonal influenza, norovirus gastroenteritis (non-enveloped virus) and rotavirus gastroenteritis (non-enveloped virus). The public health measures in order to curb the spread of COVID-19 (table 1) are indicated. [27]

Contrary to other respiratory viruses, rhinoviruses do not have an envelope. Thus, they display greater environmental stability and transmission via fomites might play a greater role. To gain insight into whether the different physicochemical properties of rhinovirus could explain its quick resurgence, we wished to assess the potential impact of NPIs on the transmission of other non-enveloped viruses, namely gastroenteritis viruses, which are not covered in our sentinel. Therefore, we reviewed aggregated data on selected viral infections reported to German public health authorities, using the *SurvStat@RKI 2.0* online tool [27]. Consistent with the sentinel observations, NPI deployment was followed by a decline in the number of reported influenza infections (Fig. 3). Interestingly, the same was true for infections with non-enveloped gastroenteritis viruses that are transmitted fecal-orally and via fomites: The norovirus winter wave, already in decline before NPI introduction, ended weeks earlier than in previous years. Similarly, rotavirus notifications, which normally peak in spring, were substantially less frequent than in 2017-2019. This implies that greater environmental stability and different transmission routes alone do not explain rhinovirus resurgence.

## 4 Discussion

In the context of the initial response to the first wave of the COVID-19 pandemic in Germany, we have observed an unprecedented decline of all respiratory viruses in our laboratory-based national surveillance system for ARIs. This decrease was temporally associated with the implementation of far-reaching NPIs, including a 4 week contact ban followed by a mask mandate. This data aligns well with the drastic decrease of notifications for a wide range of infectious diseases observed during a similar time period in Germany, which included numerous notifiable illnesses with a respiratory transmission route, including influenza [26]. It is plausible and to be expected that these findings reflect, at least in part, a true suppression of respiratory virus transmission and circulation, secondary to the public health measures implemented to curb the spread of SARS-CoV-2.

However, certain confounding factors must also be considered. For example, especially in the initial phase of the pandemic, there was a reluctance to seek medical care in the general population [28,29], potentially resulting in fewer diagnoses and notifications of respiratory virus infections. This aspect, discussed in depth by Ulrich and colleagues [26], is also of importance with respect to the sharp drop of aggregated notification data observed here for influenza and noro-/ rotavirus (Fig. 3).

Several points support the assumption that there has been a true decline in the burden of respiratory viral illness:

1. An internet-based participatory surveillance system, GrippeWeb, established at RKI in 2006 to monitor ARI activity [30], revealed that from weeks 13 to 27, the estimated weekly ARI rates were at levels well below those of previous years [31,32]. This data is provided by voluntary participants in the general population, who do not necessarily see a physician for their symptoms. Therefore, it renders additional support to the hypothesis that the decreased sentinel prevalence of respiratory viruses and decreased notifications of Influenza-/RSV- cases subsequent to NPI introduction represented indeed a true decline in respiratory virus circulation.
2. With respect to reports of other viral illnesses, these remained low even after the vast majority of restrictions had been lifted and mobility levels had returned to normal (Fig. 3) Thus, it appears less likely that reluctance to seek healthcare during the pandemic was the primary cause for decreased notifications.
3. Our findings align with observations made in other countries from both Southern and Northern Hemisphere countries regarding a premature end to the influenza season of 2019/20 [[3], [4], [5],^9^,^33^].
4. Our virological surveillance study considers sentinel prevalence (percent positive tests among patients presenting with ARI to physicians’ offices) rather than absolute numbers, a parameter that is considerably less affected by medical care underutilization [34]. In addition, our study design calls for sentinel physicians to swab patients fulfilling the ARI case definition, which has been maintained throughout. In other words, unlike SARS-CoV-2 testing criteria, which were loosened as national testing capacities increased, the formal diagnostic approach applied to sentinel patients has not changed during the observation period.

An implication of our findings, together with those of Ullrich et al. and observations from other countries [[3], [4], [5],^9^,^26^] is that NPIs, followed by a mask mandate, may have sustained impact on the circulation of respiratory viruses. This impact may be more pronounced during summer, when respiratory viral activity is low in general; however, similar effects were observed during Southern Hemisphere winter [5], [6], [7]. This implies a beneficial effect of NPIs, given the substantial morbidity and mortality not only of COVID-19 and influenza, but also other viral ARIs including RSV, one of the most important agents of severe lower respiratory tract infections in young children [35,36].

From a public health point of view, both short-term intense physical distancing measures and mask mandates should be considered as means to decrease the burden of respiratory viral illness during future winter season.

Once restrictions were being loosened, only rhinovirus appeared to resurge topre-pandemic levels, similar to findings reported in studies fromAustralia[7], England[37], Japan [2] and New Zealand [3].

In contrast to other respiratory viruses, which display distinct seasonality with clear preponderance in winter, rhinoviruses circulate throughout the year [38]. Whereas their relative prevalence decreases in winter due to influenza interference, they are by far the most prevalent respiratory viral agent during summer months [39,40]. Additionally, rhinoviruses differ from other respiratory viruses in that they are non-enveloped and thus more tenacious. The relative contribution of different transmission routes and their variations between different viruses have thus far not been determined conclusively [41,42]. It is possible that rhinovirus spread depends to a larger part on fomite transmission, which is not prevented effectively by masks. However, rhinovirus seasonality, tenacity, and potentially different transmission route do not provide a sufficient explanation for their resurgence, given that other unenveloped viruses with spring / summer peaks and potential for fomite transmission did not resurge: rotavirus, norovirus (Fig. 3) and enterovirus^4^. Moreover, a similar pattern was reported from Australia, where rhinovirus but not influenza rebounded during Southern Hemisphere winter [7]. Furthermore, a recent study indicates that face masks might be less efficient at filtering rhinoviruses out of exhaled breath than they are at reducing influenza and seasonal coronaviruses [43]; though confirmatory studies are currently lacking, this may be considered as a factor contributing to rhinovirus rebound despite universal masking, particularly in adults.

For several weeks in the 2020 summer, rhinovirus detection prevalence exceeded that noted during corresponding weeks in previous years. Similar observations have been made in New Zealand [3] and Japan [2]. Potential explanations for this phenomenon include a comparative increase in diagnostic testing, where patients presenting with relatively mild clinical symptoms, who in years prior to the pandemic would not have seen a physician, are swabbed; the substantial decreases of all other respiratory viruses leading to relative overrepresentation of rhinovirus; and a high proportion of children being tested, in whom rhinovirus infections occur frequently. In addition, cross-serotype T-cell mediated immunity to rhinovirus exists [44] and might require frequent boosting; lack of rhinovirus exposures might in turn result in waning population immunity, increased susceptibility and higher prevalence of this agent. A similar effect of NPIs - waning population immunity due to lack of exposure, ultimately leading to very high incidence once restrictions are lifted - should be anticipated with respect to other respiratory viruses and has also been projected in a recent modelling study [34]. Robust ARI surveillance and, for influenza, broad vaccination coverage should be ensured during the coming seasons.

Rhinovirus rebound was first observed in pediatric samples, which may be related to the higher susceptibility of children, who are immunologically naïve to many rhinovirus serotypes. Children are considered a natural reservoir of this viral agent, which easily spreads in daycare centers and schools [45,46]. In addition, especially small children are unable to follow physical distancing and common hygiene measures and masks are not readily available for younger age groups. Rhinovirus detection rates started rising after school / daycare reopenings, confirming a recent report by Poole et al., which suggested that rhinovirus transmission is mainly driven by children [37]. Respiratory virus transmission is generally common among children, especially in school / daycare settings [47], [48], [49]. It is important that these agents continue to be watched diligently in this age group.

One limitation to our study is that the behavioral or procedural changes driven by the pandemic or measures to control it may have influenced sentinel sampling. For example, sample counts exceeded those of the previous years almost every week beginning in week 17, 2020. A potential explanation might be heightened public awareness lowering the threshold (a) to see a physician for ARI symptoms and/or (b) to test a patient presenting with ARI symptoms for respiratory viruses. On the other hand, in 2020, a considerable fraction of respiratory virus diagnostics was performed in test centers, practices focusing on COVID-19 testing. Thus, we can not completely rule out the possibility that the composition of sentinel specimens obtained in 2020 differed from that of previous years, which might affect comparability.

In summary, we have examined a portion of the population with ARI over the course of 38 weeks in 2020, spanning the summer season following the most substantial implementation of NPIs in modern German history. Our observations indicate that NPIs in early spring, followed by a mask mandate, may be extremely effective at reducing respiratory virus circulation. With mask mandates and many physical distancing measures remaining in place influenza detections in our sentinel were virtually absent during the subsequent winter season, as our preliminary data analyses have revealed (data not shown). This indicates that influenza activity in Germany was at at historically low levels throughout the 2020/21 winter season, mirroring the observations made during Southern hemisphere winter [5,7][3]. An impressive rebound of rhinovirus, also reported from the Southern hemisphere, was noted in young children first. It may result not only from distinct virological features of this agent but also from immunological naïveté of and less physical distancing in young children. It is worth noting that rhinovirus has been the most common respiratory pathogen isolated in adults with community-acquired pneumonia, although its role is not fully understood yet [50]; and that rhinoviruses belong to one of five viral groups considered likely sources of future global catastrophic biological risk [18]. Systematic virological surveillance for these agents is currently lacking, at least on a global scale. Given their pandemic potential, building active laboratory-based sentinels targeting these and other respiratory RNA viruses is a global health imperative.

## Author contributions

DYO wrote the manuscript with support from SB, BB, JR, MvK, MM, TW and RD; SB is responsible for the sentinel system, including practice recruitment and study coordination; DYO, BB, JR, SD, MW, TW and RD contributed to data collection, study coordination and laboratory-based analyses for the virological sentinel; FS obtained and analyzed mobility data; MvK performed statistical analyses; MM, TW and RD supervised the project.

## Data sharing statement

Due to data protection regulations, individual participant data can not be made available. With respect to opportunities to collaborate, please contact the authors.

## Conflict-of-interest statement

The authors have no conflicts of interest to declare.

## Appendix Supplementary materials

Image, application 1

## Footnotes

1 https://influenza.rki.de

2 For reasons of clarity and legibility, Fig. 2 displays data from weeks 9-32. The full observation period (weeks 1-38, 2020 and weeks 1-52, 2017-2019) is covered in Supplementary Figure S2.

3 The observed one-week time lag corresponds to the usual delay between sampling date (applied to virological surveillance specimens) and reporting date (on which positive lab results are relayed to Public Health Authorities).

4 K. Keeren, personal communication on Oct. 9, 2020 (separate manuscript in preparation).
