# Supplementary material for: Trends in respiratory virus circulation following COVID-19-targeted nonpharmaceutical interventions in Germany, January - September 2020: Analysis of national surveillance data
Source: Lancet Reg Health Eur. 2021 Jun 7;6:100112. doi: 10.1016/j.lanepe.2021.100112 (PMC8183189; doi:10.1016/j.lanepe.2021.100112)
Supplement: Supplementary file 1 [file mmc1.pdf]

SUPPLEMENTARY MATERIAL

1. **Supplementary Table S1.** Modular real-time PCR assay system for the detection of respiratory viruses: Oligonucleotides.
PAGE 2-3

2. Supplementary Table S2. Virological surveillance 2017-2020: Specimens and viral pathogen distribution, weeks 1-38 (2020) and weeks 1-52 (2017-2019).
PAGE 4

3. Supplementary Table S3. Statistical test results of respiratory virus prevalence in 2020 being significantly lower than in the years 2017-2019 (weeks 1-38).
PAGE 4

4. Supplementary Figure S1. Relationship of sample numbers and minimum pathogen pre-test prevalence that can be detected with 95% probability.
PAGE 5

5. Supplementary Figure S2. Respiratory virus surveillance results in the context of public health measures, weeks 1-38 (2020) and weeks 1-52 (2017-2019).
PAGE 6-7

6. Supplementary Figure S3. Heatmap diagrams, showing the percentage of rhinovirus-positive specimens by age group (Y-axis) and time (X-axis) in weeks 1-38, 2017-2020.
PAGE 8

7. Supplementary Figure S4. Heatmap diagrams, showing the ratio of absolute numbers of rhinovirus-positive specimens over total specimens by age group (Y-axis) and time (X-axis) in weeks 1-38, 2017-2020.
PAGE 9

Supplementary Table S1.
Modular real-time PCR assay system for the detection of respiratory viruses*,: Oligonucleotides used at the German National Influenza Center.**

Assay	Assay description	Oligonucleotide Name***	Oligonucleotide Sequence (5'→3') ****	Final concentration
IV-A_M	Generic detection of Influenza A [M-segment] [1]	M+25	AGATGAGTCTTCTAACCGAGGTCG	300
		M-124sw	CTGCAAAGACACTTTCCAGTCTCTG	300
		M-124 BB	CCWGCAAARACATCYTCAAGTYTCTG	600
		MGB M+64	*TCAGGCCCCCTCAA - MGB*	100
IV-B(YamVic)_HA	Detection of human Influenza B [HA-segment] differentiation of Yamagata and Victoria lineages *oligonucleotides modified from Biere et al.* [2]	FluB HA YamVic F432	ACCCTACARAMTTGGAACYTCAGG	900
		FluB HA YamVic R479	ACAGCCCAAGCCATTGTTG	600
		B-Yam MGB437	*AATCCRMTTTTACTGGTAG - MGB*	150
		FluB HA Vic MGB470	*ATCCGTTTCCATTGGTAA - MGB*	100
HRV	HRV [5'UTR] [3]	HRV 375 F1	GTGKYCYAGCCTGCGTGGC	300
		HRV 586 R1	ACGGACACCCAAAGTAGTYGGT	300
		S-HRV 476	*CCTCCGGCCCCTGAATGYGGCTAA*	100
RSV_L	RSV A + B [L-Gene]; *oligonucleotides modified from Papillard-Marechal et al.* [4]	RSV L F	GTGGAACTTCATCCTGAYATAAGATATATT	600
		RSV L R	GTTGCATCTGTAGCRGGAATGGT	600
		RSV L MGB	*TTGCAATGATCATAGTTTACC - MGB*	100
HMPV	HMPV A+B [NP-Gene] [5];*oligonucleotides modified from Clark et al.* [6]	HMPV Clark F	GGCAAYATYCCACAAAAYCAGAG	900
		HMPV Clark R	AAGGCACCTACACATAATAARATTATDGG	900
		HMPV Clark MGB	*TCAGCACCAGACACA - MGB*	100
PIV-1-4	PIV-1 [HN-Gene]	PIV-1 F7508	TGCAATATATGCRTATTCATCAAACTTAAT	300
		PIV-1 R7587	CTAATTGTAAAACCTGATATGACTTCCCTA	300
		PIV-1 MGB7539	*ACTCAAGGATGTGCAGATA - MGB*	100
	PIV-2 [HN-Gene]	PIV-2 F7460	ATCTTCAGGACTATGAAAACCATTTACC	300
		PIV-2 R7544	CACAACCTCCTGGTATAGCAGTGAC	300
		PIV-2 TM7489	*AAGTGATGGAATCAATCGCAAAAGCTGTT*	100
	PIV-3 [HN-Gene]	PIV-3 F8271	GCATTGTATCATCTGTCATATTRGAYTCAC	600
		PIV-3 R8364	GCCAGCTCGTTYACYCTTTCRGT	600
		PIV-3 TM8306	*TCGAGAGTBAACCCAGTCATAACTTACTCAACA*	150
	PIV-4 [P-Gene]	PIV-4 F3028	AGACGTCTCAAAATTTGTTGATCAAG	300
		PIV-4 R3103	GGTTCCAGAYAAWATGGGTCTTGCTA	600
		PIV-4 MGB3085	*TCAAGTGTAATTGTATTRTC - MGB*	150
ASSAYS FOR INFLUENZA A SUBTYPING				
IV-A_pdm_HA	human Influenza A strains [HA-segment], specific detection of H1pdm09, differentiation of prepandemic H1 *oligonucleotides modified from Schulze et al.* [1]	FluA H1pdm F236	TGGGAAATCCAGAGTGTGAATCACT	300
		FluA H1pdm R318	CGTTCCATTGTCTGAAYTAGATGTT	600
		FluA H1pdm TM324	*CCACAATGTAGGACCATGADCTTGCTGTG*	150
IV-A(H3N2)_HA	Influenza A(H3N2) [HA-Segment], specific detection of human H3 *oligonucleotides modified from Schulze et al.* [1]	FluA H3 F235	CAGTCCTCATCAGATCCTTGATGG	300
		FluA H3 R333	GGTCCCATTTCTTATTTTGAAAGCC	300
		FluA H3 MGB286	*TATTGGGRGACCCTCAGT*	150
IV-A(N1)pdm_NA	Influenza A(H1N1)pdm09 [NA-segment], specific detection of human N1, differentiation of prepandemic H1N1 *oligonucleotides modified from Schulze et al.* [1]	FluA N1pdm F1255	AGACCTTGCTTCTGGGTTGAAC	300
		FluA N1pdm R1334	AAGGATATGCTGCTCCCRCTAGT	300
		FluA N1 TM1318	*CAGATTGTGTTCTCTTYGGGTCGYCCT*	150
IV-A(N2)_NA	Influenza A(H3N2) [NA-Segment], specific detection of human N2 *oligonucleotides modified from Schulze et al.* [1]	FluA N2 F769	TGATACTAAAATAYTATTCATTGAGGAGGG	600
		FluA N2 R892	GCAGACACATCTGACACCAGGRTAT	600
		FluA N2 TM804	*TCGTTCATACTAGCAMATTGTCAGGAAGTGC*	150

Assay	Assay description	Oligonucleotide Name***	Oligonucleotide Sequence (5'→3') ****	Final concentration
INTERNAL CONTROL				
FCV	Feline Calicivirus (Internal Control)	FCV F54	CGTTACCGCCACACCCAT	300
		FCV R141	GAGTTCACGAAAGATTTCAGACCAT	300
		FCV TM96	*ACCCATCATTCTAACACTCCCGCCAAT*	*100*

* Multiplex combinations are available upon request.

** For SARS-CoV-2 detection, the oligonucleotides described by Corman et al.[7] (E-Gen) and Michel et al.[8] (ORF1ab) are employed without sequence modifications.

*** Normal font indicates primers, *italics* indicate probes.

**** Sequences of oligonucleotides in use for virological surveillance are reviewed and adjusted annually, taking viral genetic evolution into account. Sequences shown here are up to date as of October, 2020. MGB probes are indicated by "- MGB" at the 3' end of the oligonucleotide sequence.

Supplementary Table S1 References.

1. Schulze M, Nitsche A, Schweiger B, Biere B. Diagnostic approach for the differentiation of the pandemic influenza A(H1N1)v virus from recent human influenza viruses by real-time PCR. *PLoS One* 2010; **5**(4): e9966.
2. Biere B, Bauer B, Schweiger B. Differentiation of influenza B virus lineages Yamagata and Victoria by real-time PCR. *J Clin Microbiol* 2010; **48**(4): 1425-7.
3. Reiche J, Bottcher S, Diedrich S, et al. Low-level Circulation of Enterovirus D68-Associated Acute Respiratory Infections, Germany, 2014. *Emerg Infect Dis* 2015; **21**(5): 837-41.
4. Papillard-Marechal S, Enouf V, Schnuriger A, et al. Monitoring epidemic viral respiratory infections using one-step real-time triplex RT-PCR targeting influenza A and B viruses and respiratory syncytial virus. *J Med Virol* 2011; **83**(4): 695-701.
5. Reiche J, Jacobsen S, Neubauer K, et al. Human metapneumovirus: insights from a ten-year molecular and epidemiological analysis in Germany. *PLoS One* 2014; **9**(2): e88342.
6. Clark TW, Medina MJ, Batham S, Curran MD, Parmar S, Nicholson KG. Adults hospitalised with acute respiratory illness rarely have detectable bacteria in the absence of COPD or pneumonia; viral infection predominates in a large prospective UK sample. *J Infect* 2014; **69**(5): 507-15.
7. Corman VM, Landt O, Kaiser M, et al. Detection of 2019 novel coronavirus (2019-nCoV) by real-time RT-PCR. *Euro Surveill* 2020; **25**(3).
8. Michel J, Neumann M, Krause E, et al. Resource-efficient internally controlled in-house real-time PCR detection of SARS-CoV-2. Virology Journal 2021; *in press.*

Supplementary Table S2.
Virological surveillance 2017-2020: Specimens and viral pathogen distribution (weeks 1-52)

Year	N	Virus detected							
		IV A/B	HRV	HMPV	RSV	PIV	SARS-CoV-2	≥2	none
**2017**	5265	1325	796	121	312	0	0	63	2648
**2018**	5886	2109	657	211	220	0	0	99	2590
**2019**	4199	1078	600	108	304	1	0	99	2009
**2020**	3580	836	495	189	152	43	12	54	1799

IV A/B, human influenza virus A/B; RSV, respiratory syncytial virus; HMPV, human metapneumovirus; HRV, human rhinovirus; PIV, parainfluenzavirus; SARS-CoV-2, SARS coronavirus 2; ≥2, more than one virus detected; 0, all PCRs with negative results.

Supplementary Table S3.
Statistical test results of respiratory virus prevalence in 2020 being significantly lower than in the years 2017-2019 (weeks 1-38) using a binomial test (bino) or a Fisher Exact test (fisher)

Week	P-Value (bino)	P-Value (fisher)
1	0.74564	0.87866
2	0.82492	0.86771
3	0.92177	0.92327
4	0.99994	0.99865
5	0.97395	0.97084
6	0.75279	0.79523
7	0.38434	0.54902
8	0.99654	0.98976
9	0.99982	0.99509
10	0.99998	0.99786
11	0.99991	0.9923
**12**	0.00886	0.11205
13	0.98253	0.92861
**14**	**5.16E-05**	**0.009879**
**15**	**0.000296**	**0.007139**
**16**	**2.38E-06**	**0.000668**
**17**	**5.34E-06**	**0.003894**
**18**	**0**	**0.001467**
**19**	**4.11E-12**	**2.70E-05**
**20**	**3.57E-11**	**7.73E-05**
**21**	**4.51E-06**	**0.004473**
**22**	**0**	**0.000167**
23	0.059752	0.39933
24	0.69068	0.88247
25	0.92014	0.96211
26	0.86921	0.94085
27	0.99999	0.9995
28	1	1
29	1	1
30	1	1
31	0.99709	0.99619
32	1	0.99995
33	0.99996	0.99989
34	1	1
35	1	1
36	0.99999	0.99966
37	1	1
38	0.99998	0.99992

Supplementary Figure S1

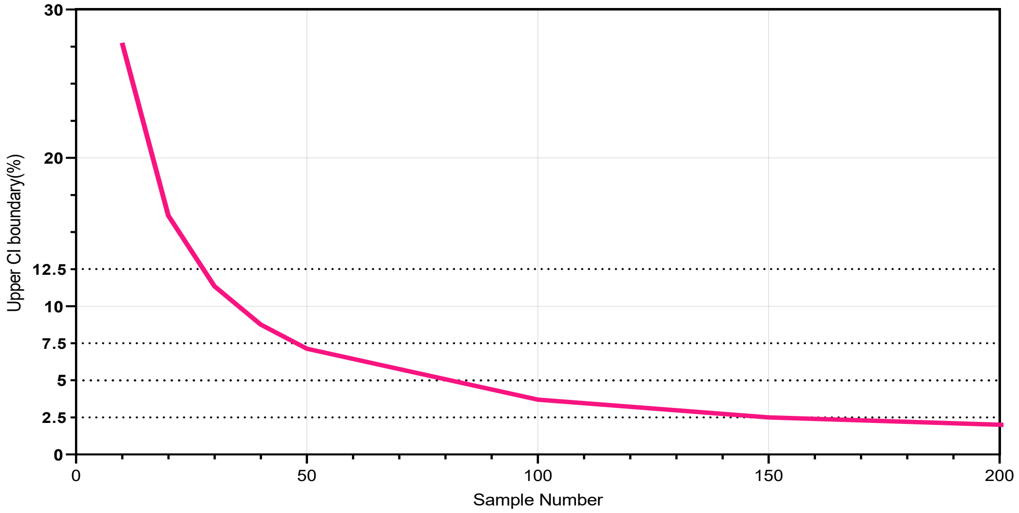

Supplementary Figure S1. Relationship of sample numbers and minimum pathogen pre-test prevalence that can be detected with 95% probability.

Supplementary Figure S2

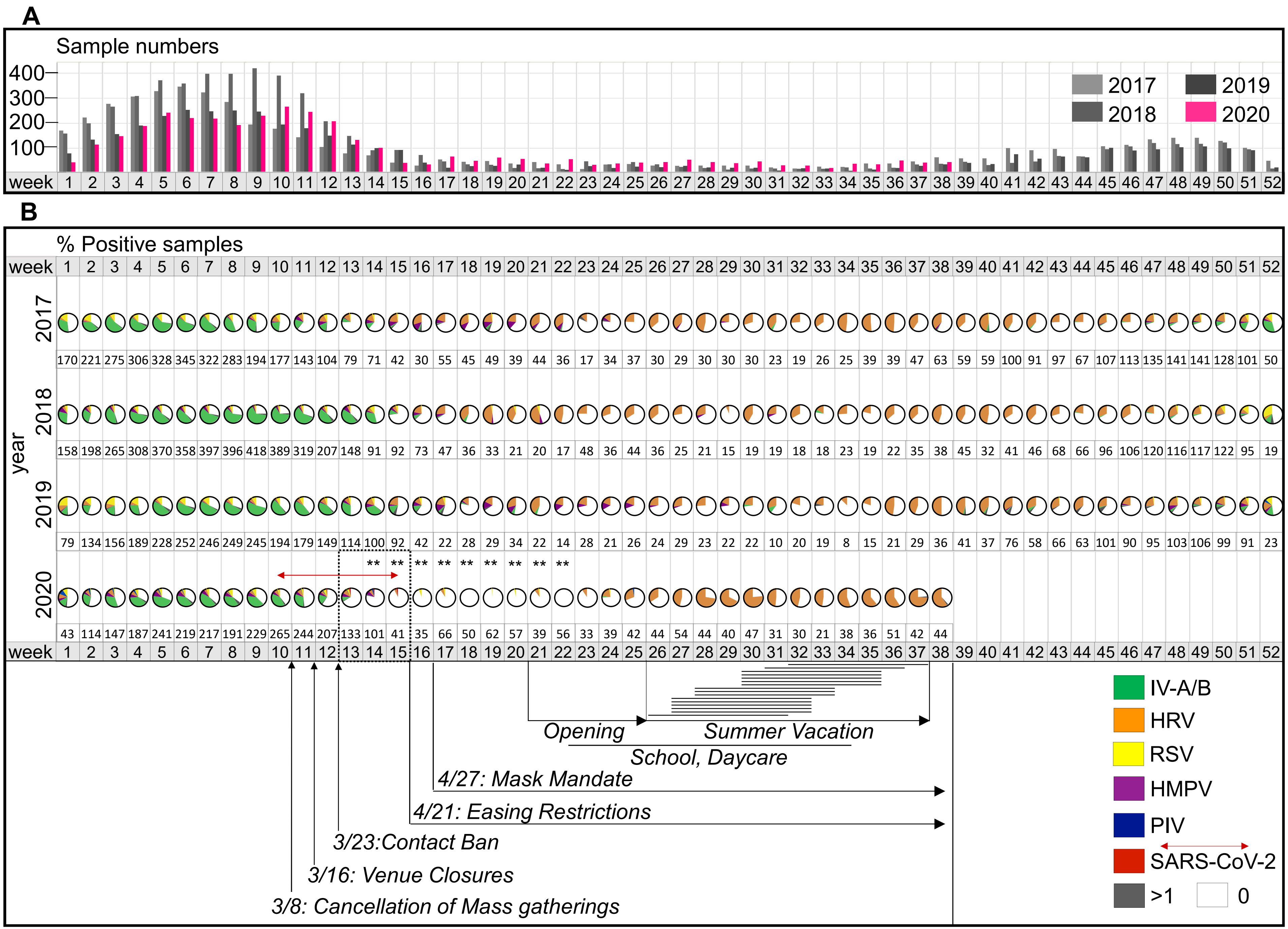

Supplementary Figure S2. Respiratory virus surveillance results in the context of public health measures.
A. Specimen numbers per sampling week/ year. Each column represents the numbers of samples obtained from patients presenting with acute respiratory illness in the calendar week indicated. Shades of grey / pink indicate the sampling year.
B. 2020 sentinel prevalence of respiratory viruses in the temporal context of NPIs and mobility, compared to 2017-2019 sentinel prevalence. Areas of colored segments in each piechart represent the detection prevalence of a respiratory virus in samples obtained during the indicated week / year. Numbers in each cell represent the N of samples obtained and examined during the indicated week / year. Included is a 2020 mobility chart, displaying the relative change [%] in population mobility as compared to the corresponding 2019 week; an overview of NPIs; and summer vacation periods, which vary by region: each horizontal staggered line represents the the six-week vacation block of one Bundesland (state). Asterisks denote statistical significance level of 2020 respiratory virus prevalence being lower than in 2017-2019, based on both binomial and Fisher's exact tests. **, $p<0.005$.
IV: Influenza A/B, HRV: Human Rhinovirus, RSV: Respiratory Syncytial Virus, HMPV: Human Metapneumovirus, PIV: Parainfluenzavirus, SARS-CoV-2: SARS Coronavirus 2, 0: negative for the tested viruses, >1: more than 1 virus detected. Both PIV and SARS-CoV-2 were only tested for in 2020.

Supplementary Figure S3

2017

Age (years)																			
>60	2%	2%	3%	1%	4%	3%	22%	20%	15%	15%	25%	0%	0%	75%	50%	0%	25%	33%	40%
35-60	5%	3%	1%	2%	7%	1%	7%	40%	12%	14%	25%	10%	40%	39%	13%	45%	46%	47%	50%
16-34	6%	7%	3%	7%	9%	13%	0%	11%	38%	57%	62%	25%	20%	40%	31%	44%	25%	64%	44%
5-15	2%	0%	3%	5%	18%	13%	10%	17%	22%	20%	20%	0%	25%	17%	0%	0%	36%	60%	47%
<4	10%	11%	7%	7%	18%	34%	29%	32%	31%	29%	41%	33%	33%	50%	31%	38%	47%	46%	24%
**week**	1-2	3-4	5-6	7-8	9-10	11-12	13-14	15-16	17-18	19-20	21-22	23-24	25-26	27-28	29-30	31-32	33-34	35-36	37-38

2018

Age (years)																			
>60	11%	2%	4%	2%	1%	1%	3%	18%	20%	40%	100%	13%	33%	20%	0%	0%	0%	0%	33%
35-60	4%	3%	4%	4%	1%	2%	5%	8%	22%	22%	50%	42%	46%	33%	44%	25%	22%	33%	50%
16-34	9%	4%	8%	8%	5%	6%	5%	14%	44%	36%	60%	50%	41%	71%	20%	25%	33%	30%	44%
5-15	8%	4%	2%	2%	2%	4%	11%	15%	33%	29%	29%	14%	22%	0%	17%	20%	14%	13%	10%
<4	8%	11%	10%	10%	12%	12%	15%	21%	33%	63%	40%	15%	25%	19%	33%	47%	23%	42%	50%
**week**	1-2	3-4	5-6	7-8	9-10	11-12	13-14	15-16	17-18	19-20	21-22	23-24	25-26	27-28	29-30	31-32	33-34	35-36	37-38

2019

Age (years)																			
>60	13%	6%	6%	4%	3%	5%	0%	15%	11%	17%	0%	25%	0%	40%	33%	0%	n/a	0%	100%
35-60	15%	9%	4%	5%	7%	9%	9%	7%	25%	25%	43%	38%	14%	38%	9%	14%	0%	13%	57%
16-34	13%	12%	7%	5%	8%	7%	9%	29%	0%	17%	50%	25%	60%	18%	30%	67%	0%	40%	67%
5-15	6%	6%	9%	2%	3%	13%	10%	27%	14%	27%	33%	19%	15%	18%	14%	20%	40%	63%	10%
<4	13%	11%	14%	10%	5%	13%	22%	33%	26%	29%	29%	8%	23%	25%	23%	21%	31%	36%	44%
**week**	1-2	3-4	5-6	7-8	9-10	11-12	13-14	15-16	17-18	19-20	21-22	23-24	25-26	27-28	29-30	31-32	33-34	35-36	37-38

2020

Age (years)																			
>60	0%	14%	6%	2%	9%	5%	3%	0%	0%	0%	8%	0%	20%	0%	50%	50%	0%	20%	33%
35-60	8%	8%	3%	7%	8%	5%	4%	0%	0%	0%	0%	0%	13%	46%	50%	50%	0%	40%	56%
16-34	12%	12%	7%	7%	5%	10%	10%	0%	7%	0%	0%	0%	18%	29%	83%	67%	63%	50%	63%
5-15	0%	5%	3%	1%	4%	4%	9%	0%	3%	0%	0%	23%	21%	66%	75%	27%	61%	68%	71%
<4	8%	12%	8%	5%	8%	14%	19%	8%	5%	0%	5%	26%	44%	64%	75%	53%	54%	63%	77%
**week**	1-2	3-4	5-6	7-8	9-10	11-12	13-14	15-16	17-18	19-20	21-22	23-24	25-26	27-28	29-30	31-32	33-34	35-36	37-38

Supplementary Figure S3. Heatmap diagrams, showing the percentage of rhinovirus-positive specimens by age group (Y-axis) and time (X-axis) in weeks 1-38, 2017-2020. Each heatmap diagram corresponds to the indicated calendar year. Time scale by two-week-blocks as indicated. The corresponding absolute numbers are provided in Supplementary Figure S4.

Supplementary Figure S4

2017

Age (years)																			
>60	1/58	2/82	3/113	1/114	2/57	1/31	4/18	1/5	2/13	2/13	3/12	0/4	0/1	3/4	5/10	0/1	1/4	3/9	2/5
35-60	6/118	6/175	2/211	4/195	7/97	1/67	3/43	6/15	2/17	2/14	4/16	1/10	6/15	9/23	2/15	5/11	6/13	9/19	12/24
16-34	4/72	8/116	4/120	9/121	6/66	4/31	/17	1/9	5/13	4/7	8/13	2/8	3/15	4/10	4/13	4/9	1/4	7/11	11/25
5-15	1/50	0/107	4/120	4/88	9/51	7/54	3/29	2/12	4/18	4/20	2/10	0/11	3/12	1/6	0/6	0/8	4/11	9/15	9/19
<4	9/94	11/101	8/109	6/86	18/100	22/64	12/42	10/31	12/39	10/34	12/29	6/18	8/24	8/16	5/16	5/13	9/19	11/24	9/37
**week**	1-2	3-4	5-6	7-8	9-10	11-12	13-14	15-16	17-18	19-20	21-22	23-24	25-26	27-28	29-30	31-32	33-34	35-36	37-38

2018

Age (years)																			
>60	6/55	1/62	4/105	2/118	1/119	1/72	1/35	4/22	2/10	2/5	2/2	1/8	2/6	1/5	0/5	0/5	0/3	0/2	1/3
35-60	5/117	5/186	9/256	10/283	3/281	3/157	4/82	4/53	4/18	2/9	4/8	8/19	11/24	4/12	4/9	1/4	2/9	3/9	10/20
16-34	5/58	4/90	10/120	12/142	7/137	5/84	2/40	3/22	7/16	5/14	3/5	8/16	7/17	5/7	1/5	2/8	3/9	3/10	7/16
5-15	4/52	4/112	2/119	3/123	2/132	4/97	3/27	3/20	5/15	2/7	2/7	2/14	2/9	/6	1/6	1/5	1/7	1/8	1/10
<4	6/74	13/123	12/126	13/126	16/138	14/115	8/55	10/48	8/24	12/19	6/15	4/27	6/24	3/16	3/9	7/15	3/13	5/12	11/22
**week**	1-2	3-4	5-6	7-8	9-10	11-12	13-14	15-16	17-18	19-20	21-22	23-24	25-26	27-28	29-30	31-32	33-34	35-36	37-38

2019

Age (years)																			
>60	5/40	2/35	4/70	2/57	2/58	2/42	0/20	2/13	1/9	1/6	0/3	1/4	0/3	2/5	1/3	0/1	0/0	0/1	2/2
35-60	8/54	9/100	5/135	8/152	10/151	8/88	5/55	2/29	2/8	3/12	3/7	3/8	1/7	5/13	1/11	1/7	0/5	1/8	8/14
16-34	6/46	6/52	6/85	5/104	6/74	4/55	3/33	5/17	0/7	1/6	3/6	2/8	3/5	2/11	3/10	2/3	0/3	2/5	6/9
5-15	1/18	3/54	6/70	1/66	2/61	7/54	4/42	8/30	1/7	3/11	2/6	3/16	2/13	2/11	1/7	1/5	2/5	5/8	1/10
<4	9/72	11/101	16/116	12/116	5/95	11/87	14/64	15/45	5/19	8/28	4/14	1/13	5/22	3/12	3/13	3/14	4/13	5/14	12/27
**week**	1-2	3-4	5-6	7-8	9-10	11-12	13-14	15-16	17-18	19-20	21-22	23-24	25-26	27-28	29-30	31-32	33-34	35-36	37-38

2020

Age (years)																			
>60	0/10	5/35	3/51	1/48	5/56	3/56	1/38	0/10	0/15	0/9	1/12	0/4	1/5	0/2	1/2	1/2	0/2	1/5	2/6
35-60	4/50	7/91	4/133	8/114	12/154	7/132	3/68	0/16	0/20	0/27	0/17	0/11	1/8	6/13	4/8	3/6	0/3	2/5	5/9
16-34	3/26	6/52	5/75	5/68	5/95	9/94	4/39	0/10	1/15	0/13	0/6	0/12	2/11	2/7	5/6	2/3	5/8	4/8	5/8
5-15	0/15	3/62	3/95	1/68	3/76	3/72	3/35	0/16	1/29	0/27	0/22	3/13	4/19	19/29	15/20	4/15	11/18	19/28	20/28
<4	3/37	11/89	8/106	6/110	9/111	13/95	10/52	2/24	2/37	0/42	2/38	8/31	19/43	30/47	38/51	18/34	15/28	26/41	27/35
**week**	1-2	3-4	5-6	7-8	9-10	11-12	13-14	15-16	17-18	19-20	21-22	23-24	25-26	27-28	29-30	31-32	33-34	35-36	37-38

Supplementary Figure S4. Heatmap diagrams, showing the ratio of absolute numbers of rhinovirus-positive specimens over total specimens by age group (Y-axis) and time (X-axis) in weeks 1-38, 2017-2020. Each heatmap diagram corresponds to the indicated calendar year. Time scale by two-week-blocks as indicated. Specimens with unclear PCR-results on rhinovirus testing were excluded from this analysis.
